# Provincial Medical Association

**Published:** 1839-10

**Authors:** 


### PART FOURTH.

### Medical Intelligence.

---

### PROVINCIAL MEDICAL ASSOCIATION.

The Seventh Anniversary Meeting of this Society was held at Liverpool, on Wednesday and Thursday, the 24th and 25th July; and exceeded every preceding one, both in the number of members present and the interest excited by the various proceedings that took place. We regret that we are compelled, by want of room, to give so imperfect a report of this important meeting.

In the forenoon of Wednesday the members of the Council and the various Committees met to transact business at the Medical Institution. At three o'clock the General Meeting took place in the Theatre of the same institution. Dr. Barlow, the late president, having resigned the chair to Dr. Jeffreys, the proper business of the Association began. The Secretary first read the Report of the Council, which was adopted, and ordered to be printed.

Dr. Barlow then read the Report of the Committee appointed to watch over the interests of the profession, which, as it is a document of importance, we shall give in full, together with the Petition to Parliament, unanimously adopted, after considerable discussion and some modification of the original terms.

At eight o'clock in the same evening the members again assembled, when Mr. James, of Exeter, read a report on the Progress of Surgery since 1836, which occupied two hours in the delivery. It was listened to throughout with profound attention, and elicited frequent marks of approbation. It was clearly and elegantly written, and was of an exceedingly interesting nature to surgical students. We shall have another opportunity of noticing this important document.

Mr. Turner, of Manchester, then brought forward a motion, in consequence of the recent law of the Royal College of Surgeons of London respecting hospital attendance in the large provincial towns, by which regulation it would seem that the advantages of these institutions as schools of practical surgery are not appreciated as they ought to be by the Council of the College. Mr. Turner maintained that it was not just in them to place a barrier to the success of provincial schools, by demanding a longer attendance upon provincial hospitals than upon those in the metropolis, as qualifying for their diploma; and concluded by moving that a memorial be presented to the College of Surgeons, embodying the views of the Association. After considerable discussion the motion was agreed to.

On Thursday the members breakfasted together in the great room in the Adelphi; and many of them afterwards visited the Botanical Garden, accompanied by Dr. Dickinson, the professor of Botany.

At twelve o'clock the members again assembled at the Medical Institution, when several interesting practical communications were made, and a still greater number postponed, for want of time.

Dr. Symonds, of Bristol, then read the retrospective address, on Medicine, which occupied upwards of two hours in the delivery. The learned author was much applauded during its progress, and also at the conclusion. It certainly is not inferior to any of its predecessors; and we regret that our limited space will not allow us to insert it, containing as it does a very admirable detail of all the events of importance to the profession which have occurred during the past year, and a well-digested sketch of all recent researches in medicine and the collateral sciences. The whole production was alike distinguished by sound philosophical principles, enlarged views of science, and a refined taste. When printed, it will be read with interest and advantage by the whole profession.

At the conclusion of the address, Dr. Gibson, Professor of Surgery in the University of Philadelphia (who was present) was, on the motion of Dr. Forbes, unanimously elected an honorary and corresponding member.

Dr. Conolly, of Cheltenham, then read the report of the Benevolent Fund Committee, which, although it was of a satisfactory nature, and pointed out many cases in which deserved relief had been afforded through its means, yet hardly gave such a record of subscriptions and expenditure as might be expected from so large an Association. The amount raised last year was only 50*l.*; 30*l.* of which were contributed by ladies. The report contained a well-grounded appeal to the humanity of the profession.

Dr. Cowan, of Reading, then read his report on Quackery, which we shall give at length, if our space permits.

Dr. Baron, of Cheltenham, then brought forward the report on Vaccination, which occupied three hours in delivery. It was such as was to be expected from the distinguished author of the Life of Jenner. As it was fraught with matter of the utmost importance, we trust no time will be lost in laying it before the public. We shall insert the petition to Parliament, with which it concluded, and which was unanimously adopted by the meeting.

After the conclusion of the business, the members and their friends, between three and four hundred, dined together at the Town hall; and the evening concluded with a *conversazione*, in the splendid drawing-room of the same mansion. On this occasion Mr. Ceeley, of Aylesbury, exhibited drawings of the interesting results of the experiments by which he has succeeded in inoculating the cow with small-pox.

It was settled that the next meeting of the Association should be held at Southampton; and Dr. Steed, of that town, was elected President for the ensuing year. Dr. Forbes was appointed to deliver the Anniversary Address,* and Mr. Dodd, of Chichester, to draw up the Surgical Report.

The following are the only documents laid before or emanating from this meeting, which we can find room for.

### I. *Address of* Dr. Barlow, *on resigning the Chair.*

Gentlemen: In retiring from the office which, through your kind appointment, I have held for the past year, I shall delay the proceedings of the day for only a very few moments; for there is so much necessary business to engage your attention, that I should be inexcusable if I were needlessly to trespass on time required for other purposes, and which can be so much better employed. Each year brings some increase of the business to be done at our anniversary meetings, and in the prospectus of this year's proceedings you may perceive that more hours are allotted for conducting them than on any former occasion. In our earlier meetings the whole business was transacted with ease in a single day; now two days scarcely suffice. So fully, also, do the provisions made by our constitution, with the arrangements of the Council and Secretaries, embrace all that we can beneficially attend to, that any lengthened address from me, on the present occasion, would be as superfluous as it would be ill-timed. Nevertheless, gentlemen, I cannot take my leave of you without a few parting words. And first, I must beg to return you my sincere thanks for the high honour conferred on me in being placed, even for the brief period of a year, at the head of this numerous and most important Association. To be so distinguished is an honour of which the most ambitious might be proud. If "*laudari a laudato viro*" be deemed the highest praise that can be conferred,—to hold, even for a season, the rank of "*primus inter pares*" is a distinction still higher; the one emanating from an individual judgment, the other being awarded by the collective voice of a whole community. That I have been deemed worthy of holding this rank is the highest reward of professional labours that could have been bestowed on me; for of professional merit the profession alone can adequately judge. Their approval it is which gives real eminence; and to merit their favorable opinions should be the high aspiration of all whose ambition is pure and rightly directed. For the favour conferred on me in having been appointed your President, and for the personal kindness which I have in-

* This will now be delivered by Dr. Scott, of Liverpool; Dr. Forbes having given it up.

variably experienced in my intercourse with the members of the Association ever since it was first established, I beg leave to return to each and all my sincere and heartfelt thanks. I must detain you yet, gentlemen, for a single moment, while I offer my cordial congratulations on the rapidity with which our Association advances, and the elevation which it has attained. There must be some intrinsic worth in an institution which can unite so many intelligent and reflecting minds,—which can congregate annually such numbers as attend our anniversaries, withdrawing them from arduous duties which we all know are of a nature not to brook interruption of any kind,—which can inspire so much zeal and excite so much endeavour as our brief career throughout displays. Seven years ago we commenced with about fifty members: we now number in ranks nearly twelve hundred. It is impossible but that a body so numerous, so intelligent, and so united, must henceforward exert a powerful influence over all that relates to the science or practice of physic. It will, I trust, ever be our care to ensure that this influence be directed to praiseworthy ends; and that whatever measures we may find it necessary to pursue for attaining and establishing our own just rank in the political system, shall have the well-being of the community for their ultimate aim. In retiring from the chair, gentlemen, it is with peculiar satisfaction that I resign it to one so qualified to do honour to it as your respected President elect, Dr. Jeffreys. It is not in Liverpool that I need descant on his merits, his unceasing endeavours to promote the diffusion of knowledge being here, at least, well known; but they are not unobserved, even in places far distant; and the public voice, which rarely fails to award the meed of just praise to talents and energies devoted with sincerity and zeal to the public good, will assuredly enrol in the splendid list of public characters eminent for literature and science, of which Liverpool may proudly boast, the name of my valued friend and respected successor in this chair, Dr. Thomas Jeffreys. To him I now relinquish it, with my best wishes for his welfare and happiness, and for the increasing and enduring prosperity of the Association over which he is about to preside.

### II. *Address of* Dr. Jeffreys, *on taking the Chair.*

Gentlemen: Whatever may have been the motives which gave rise to the first idea of inducing men of kindred feelings and pursuits to promote and establish meetings for the purpose of imparting to each other views and opinions conducive to their happiness and best interests as a body, there can be but one opinion as to the effect of the Association whose Seventh Anniversary we are met to celebrate. Its beneficial results reflect much lustre on those under whose direction it was originally formed. Perhaps none exceed in zeal and energy the class of individuals I now address. Amidst personal privations from limited pecuniary resources, and difficulties arising from prejudices, often sufficient to weaken the exertions of the most ardent mind, we find the medical man ever ready cheerfully to promote every good work, encountering danger and obstacles of such magnitude, that the attempt to surmount them would, from dull understandings or hearts dead to the purest and most ennobling of human affections, incur only the reproach of rashness or infatuation. The Provincial Medical and Surgical Association has been one of the very first, by personal intercourse, by the exertions of its members, and by its publications annually issuing from the press, to grapple with these difficulties, and in a manner which has "grown with its growth and strengthened with its strength," so as to justify no cold and qualified encomium, but one far surpassing the measure of my humble abilities to bestow. This truth may be judged of by the alacrity with which the members have responded to the summons of this the seventh anniversary meeting; and there cannot be a better criterion of the interest which the resources of our annual reunion can call forth, than the numerous assemblage I see around me. It is difficult to say whether the gratification is most enjoyed by the original promoters of the Association, or by those who have more recently joined it; the latter, however, equally share the fruits of our labours. It is scarcely to be expected that the seeds of science and literature can be so abundantly disseminated in this emporium of commercial greatness, as in collegiate cities; but I am inclined to think that when the spark of ignition is once fairly kindled, it will burst forth into a brilliant and lasting flame. The example of six successive years, the manner in which the Association has been received, and the complete gratification visiting members have experienced, if I may be allowed to judge from my own feelings, ought to, and I am confident will, fill the hearts and minds of my medical brethren here with satisfaction; and we must be proud that Liverpool should be selected before other places conspicuous for the illumination of the understanding, for the spread of those truths which advance the progress of knowledge and promote the welfare of society. I well know that hints have been thrown out, and apprehensions entertained, that the Association coming among us would only discover the poverty of our resources and our inferiority, when compared with the advanced state of science and progress of improvement in other places; but I say, if this be really the case, which I hope I may be allowed to doubt, the step we are now taking is the way to render the reverse the truth. It is true we cannot grant classical honours, nor display a long list of names illustrious in the annals of letters, such as are to be found in our academical retreats; but we can evince an honest and well-directed zeal; and so long as we can remember a Roscoe, a Currie, a Brandreth, an Allanson, and a Park, now all resting in the silent tomb, it would be unpardonable in us not to feel proud of their talents and virtues, which, in spite of many disadvantages, enabled them to fill so large a space in the eye of their contemporaries; and the memory of which will, I trust, lead the rising energies of our youth to emulate their worth, and to improve science as they did. It is thus that Liverpool will take an honorable station among the places visited by the Association, and her sons claim an equal share in the friendship of others, where we may in future be minded to meet. The visit of the British Association to this town was a novelty to which it will ever be pleasing to revert, not only as being the means of introducing us to the acquaintance of many persons of the first scientific and literary eminence, but of permitting us to witness their admiration of the many interesting objects our northern metropolis presented to their notice. Should such be the result of this meeting also, how grateful will it be to myself personally, and to the gentlemen associated with me in the present arrangements! From the first dawn of my professional career it was my firm belief and conviction that the mainspring of all professional attainments rests solely with the individual himself, and that no position can be honorably and satisfactorily retained without feeling the conscientious and powerful stimulus of a deep and awful responsibility. On this solid foundation must the noble edifice of science be raised. To no station or pursuit in life does this maxim more strongly apply than to the medical profession; for our conduct should ever be such as to defy the most searching scrutiny, and court the most public investigation. An equally if not more difficult task to perform is it, to disperse those delusions of empiricism, by which so many, not only of the lower but of the higher classes of the community, are led astray. But whoever acknowledges the foregoing principle to influence his conduct, must further confess a principle, which, blended with mental cultivation, is so efficacious to the formation of the medical character, and is only to be derived from that Supreme Being, who hath alike formed the minutest creature, and regulates the mind of him who sounds all the depths and heights of science. Surely if He watches with parental care the lives of all his creatures, he will protect and guide the men who go forth among their fellows to execute the purposes of his benevolence, in dispensing the blessings of health and soothing the severities of pain. "Let him therefore who reads and observes, never forget that in all this, as in everything else, there is nothing casual, nothing purposeless, nothing undesigned, and that good ends have been intended, as good purposes have been effected; and that all creation presents to him who will examine it, the incontrovertible proofs of a Great Artist, intending, designing; perfect in wisdom, absolute in power." When I look back to the very able manner in which the deservedly distinguished men who have preceded me in this chair have displayed the worth and usefulness of our Association, I am relieved from all copiousness of illustration and cogency of enforcement on these subjects. My learned and zealous friend and predecessor, Dr. Barlow, has most justly said, that "the main objects for which we are associated, as stated in our fundamental constitution, are the advancement of medical science, and the maintenance of the honour and respectability of the profession." These objects have never been neglected; and each succeeding year has afforded additional proof of the soundness of the principles on which they were based. With harmony and unity of feeling and purpose, let the good of mankind stimulate all our endeavours. We shall thus have our reward in the approbation of all such fellow-labourers who are wise, and great, and good; and this after many of us shall have terminated our earthly career.

I should not stand acquitted to myself were I not to seize this public opportunity of expressing my approbation of the able and courteous manner in which my predecessor filled his office at our last meeting at Bath, calculated indeed to inspire no common feeling of respect and gratitude; and I am sure that you will one and all pardon me if I indulge in expressing the deep debt of gratitude we owe to our talented, zealous, and indefatigable Secretary, Dr. Hastings. Nothing I can say will add to his justly-established reputation, yet I cannot deny myself the gratification of giving utterance to the sentiments which I entertain in common with the members of the Association. The times in which we live are peculiar, and consequently influence every class of men—the profession to which we belong as well as others. It is not my business to descant upon the novelties and improvements of the day, that being the province of our learned and accomplished associate, Dr. Symonds, of Bristol, who will deliver the retrospective address; yet I cannot forbear quoting the remark of one of our most eminent honorary members (Sir Astley Cooper,) whose anxiety to be with us on the present occasion was expressed to me in very warm terms, and who lately observed to me, that "although science has advanced, men have retrograded. Where (said he) is our Newton, our Herschel, our Hunter, our Davy, our Priestley, our Black, or our Scheele? This (he adds) is the age of application, not of discovery." Let us then profit by that great and honest man's remark, on a subject so consonant to his favorite habits of study; and make use of the means which Providence has placed in our hands, by practically and more extensively applying the resources which a body of 1100 or 1200 individuals can command, and which has not inaptly been denominated "The Parliament of the Profession." Much has already been done, but much, even at this time, remains untouched; and this I am confident can only be effected by the concentrated talent, zeal, and vigorous industry, which I believe belongs to this enlightened and powerful body.

[After some further interesting observations on the progress of medicine, &c., including an affectionate biographical notice of the late Dr. Rutter, of Liverpool, Dr. Jeffreys concluded:]

I have now to bring this brief and I fear imperfect part of my duty to a conclusion, anxious to avoid prolixity and infringing upon the more important business of the meeting. The increased number of interesting subjects for discussion, the extent and demands of our connexions, both foreign and domestic, with the weight of matter for deliberation, will occupy so much time, that two days will prove scarcely sufficient. No one, I apprehend, can meditate dispassionately upon the objects of this Association without being alive to the power which it has of contributing to individual pleasure, professional advantage, and universal good: there may be "a faulty spoke in the wheel of every great machine," which will afford matter for comment to those who are prone to hypercriticism; but such minor points sink into insignificance, considered with the more extended views and prospects of beneficial results; nay, it is better that in our efforts we should disregard these minor imperfections, than that the mainspring of our intentions should be disturbed, and our views not fully and happily confirmed. I have now to request you will accept the acknowledgments of the local members for the honour you confer upon the town by your presence. No entreaty should ever have induced me to undertake the responsibility which awaits the individual who addresses you from this chair, had it not been from a thorough conviction of the advantages to be derived from the efforts of the Association, not only to the profession to which I have always been zealously devoted, but also to any community which shall possess the advantage of its presence.

I beg you will accept my expressions of gratitude for the attention with which you have favoured me upon this commencement of my official duties. I am fully sensible that neither my pretensions nor my claims have entitled me to this honour. Permit me to return my most heartfelt thanks to my medical brethren of the town, who are fellow-members, to the general Council, and to the members in ordinary, for the distinguished position in which they have placed me, rendering this the very proudest day of my professional life; and when the hour of separation arrives, when each of you must return to your professional duties at home, in the same proportion that I estimate the honour done to Liverpool and myself by this visit, will be the sincere regret with which I shall have to say—*valde vale!*

### III. *Report of the Committee appointed to watch over the Interests of the Profession.*

At the anniversary meeting of the Association, held in Cheltenham, in 1837, a Committee was appointed "to watch over the interests of the profession at large," the duty specially assigned to its members being "to suggest to the Council from time to time such measures as may appear to them necessary to meet circumstances as they arise."

The terms of the appointment show that no direct obligation was imposed on this Committee to report its proceedings to the general meeting, its express duty being to offer suggestions to the Council. Nevertheless, as such appointment generally implies an obligation to report, your Committee deem it a proper respect on their part to submit to the Asociation the views which they take of the important trust confided to them. In the first place, being deputed "to watch over the interests of the profession at large," they considered that their attention was to be directed to the fundamental interests which involve the well-being of all ranks of the profession rather than to any of a subordinate nature, or to causes of complaint affecting particular departments only; and they adopted this view the more readily from the conviction which they entertain, that until the whole profession be organized on sound principles, and founded on a stable basis, no department of it can be well or effectively governed. While your Committee, however, have cordially concurred in the expediency of their own appointment, and have earnestly desired to fulfil to the best of their ability the trust reposed in them, the circumstances under which they entered on their duties have hitherto restrained all active discharge of them, the suitableness of any interference on their part being dependent on measures beyond their control, and the issue of which it was incumbent on them to await.

It must be in the recollection of all the members that the claims of the profession for an investigation of its manifold evils and imperfections were recognized by Parliament in 1834, and that on the 11th of March in that year, a Committee of the House of Commons was appointed "to enquire into and consider the laws, regulations, and usages regarding the education and practice of the various branches of the medical profession in the United Kingdom." This Committee commenced its labours on the 13th of March, and pursued its scrutiny minutely and diligently for several months. On the 13th of August a resolution of the House of Commons ordered "That the Committee have power to report the minutes of the evidence taken before them, together with their observations thereon." In compliance with this order, three successive volumes of the minutes of the evidence were laid before the House and printed, containing respectively the evidence relating to the London College of Physicians, the London College of Surgeons, and the Apothecaries' Company. Further evidences to at least an equal extent were obtained from the provincial medical faculty of England, from the Universities, and from the respective medical professions both of Scotland and Ireland. But these, though anxiously desired and expected by the profession, and by all the friends of rational reform, have never yet appeared. Why their publication has been so long delayed your Committee have been unable to ascertain. It was a probable cause of delay that the fire which consumed the Houses of Parliament in October, 1834, might have so damaged the records of the Committee of Medical Enquiry as to create a difficulty in continuing to publish uninterruptedly the further minutes. But this cause has never, so far as your Committee are aware, been pleaded; and they are, consequently, still ignorant why the remaining evidences have been withheld.

Your Committee, at the time of their appointment, confidently expected, not only that the remaining evidences of the Parliamentary Committee would speedily appear, but that, agreeably to the resolution of the House of Commons, this Committee would lay before the House a Report of their observations on the minutes; and your Committee were at the time further led to believe that on the basis of this Report, bills would be framed and introduced into Parliament for giving to the collective profession that political organization which both its own interest and those of the community at large imperatively demand. Awaiting this Report, and the announcement of the legislative measures which there was every reason to believe would be founded on it, your Committee felt that until the Parliamentary Committee should comply with the order of the House of Commons, by producing the whole of the evidences taken before them, and furnishing this Report, there was no likelihood of your Committee being able to promote the interest of the profession by any suggestions which they could offer to the Council, on which account they suffered the general meeting of last year to pass over without submitting to the Association any report of their views or intentions. Nearly five years having elapsed however since the Parliamentary Committee closed its investigations, your Committee have at length relinquished all expectation that either the unpublished minutes or a Report will be forthcoming, and they consequently deemed it their duty to institute enquiries as to whether any other course of proceeding than that which they had so long contemplated as the most appropriate and most feasible could be resorted to for accomplishing and expediting the reforms of the profession so greatly needed; and they have great satisfaction in announcing that a way has opened for effecting the desired ends far more favorable than they could have expected or hoped.

There are two modes by which, speculatively, an effective reform of the profession might be attempted; either by first organizing the profession so as to determine specially its classes and gradations, and then, in conformity with the system thus established, devising suitable qualifications for whatever departments the collective profession should comprise, leaving the special qualifications of each branch to be regulated by the government under which it should be more immediately placed; or by beginning with qualification, and by founding on it the gradations which should be recognized in a well-regulated profession. The first mode would have been that pursued, if the Parliamentary Committee had completed their enquiry so far as to furnish a Report, and had then introduced bills into Parliament for effecting the reforms which their Report should recommend. Attentive and mature consideration, however, has satisfied your Committee that the second mode is far preferable, attaining its ends with much more ease and far greater effect: and your Committee rejoice to add that the opportunity of promptly following out this second course is now happily furnished in a way which your Committee could not have anticipated, and through an instrumentality the creation of which they cannot but deem a most auspicious coincidence.

The foundation of the University of London must be well known to all the members. It being the province of this University to grant degrees in the faculties of law and of medicine, as well as in arts, one of its earliest measures was to determine the curricula of studies which should qualify candidates for obtaining the respective degrees; and in doing so, the Senate of the University proceeded in the most direct and judicious way, appointing a Committee of each faculty to devise the most suitable course of studies for each. The Committee of the faculty of medicine, after diligent and laborious consideration, sketched a Report which, in the first instance, they circulated widely among teachers and practitioners, in order to elicit the various opinions and criticisms which the document was calculated to call forth.

Having thus collected numerous opinions and comments, the Committee next circulated these, with a view to their being still more maturely considered. No more suitable mode could well be devised for bringing every topic involved under general and searching scrutiny. Having digested the several replies, the Committee next proceeded to modify their original sketch; but ere they submitted to the Senate their amended Report, this was once more laid before the several medical schools of England, Scotland, and Ireland. The Senate then proceeded to revise, with great care, the Report of the Medical Committees; and the "regulations" of the Senate, founded on this Report, are now in the possession of every medical school in the kingdom.

Your Committee must here solicit the attention of the members to some considerations connected with these regulations, which a cursory inspection of them might fail to suggest. To a casual observer, or on a transient examination, the "Regulations" of the University of London might appear, in the course of studies prescribed, to serve no other end than to create a new species of medical practitioners, and thus merely to introduce one more variety into a body in which discrepancy and inequality of qualification have long constituted a prominent evil. Were no other end contemplated, the course of studies thus enjoined, however perfect or superior to existing usages, would be utterly valueless, adding to the prevailing confusion, instead of superseding or diminishing it. But the views of the framers of the Medical Report were far from being so limited or misdirected. Impressed with the great and manifold evils which result from the present complicated and incongruous state of the profession, it appears to have been their design so to frame their Report that it should serve as a foundation for the legislative enactments necessary for establishing uniformity of qualification throughout the kingdom. In providing an improved qualification for the individual practitioner, they prepare the way for the still higher object of ensuring perfect competency in the entire body, and in every department; and, with a singular felicity, the scheme of reform typified in the "regulations" of the University, not only provides full security for every member introduced into the profession being competently qualified, but it consolidates the whole profession, restoring its natural unity, without disarranging those distinctions in practice which time and the natural tendencies of the profession have insensibly created. In this view the Committee of the Medical Faculty, deputed by the Senate of the University of London, have been acting, not merely as projectors of a curriculum of studies, but as profound and effective reformists of the whole profession. According to their plan, the Bachelors of Medicine would be the general practitioners of the kingdom, qualified by their education to practise every branch of the art. The Bachelors, who should look only to obtaining a legal qualification to practise with public attestation of their competency, would be content so to remain; while the more ambitious would advance to the degree of Doctor. The signal excellencies of this scheme become manifested in proportion as it is scrutinized and followed out into its practical operations. In the first place there would be the great advantage of every member entering the profession being fully attested, both as to his general and professional acquirements; and thus the best security would be afforded for the profession continuing to maintain the eminence, literary, scientific, and moral, so long accorded to it. Secondly, to every member the way would be open for reaching the highest distinction, according as talents or inclination might prompt. The more advanced distinction being given not in lieu of the primary, but in addition to it, each class would understand its own position, and no unseemly rivalry could be engendered. If a feeling of honest pride or other motive should at any time incline a Bachelor to become a Doctor, it would be always in his power, by a slight effort, so to do. Thus the system would realize what has ever proved the most powerful incentive to human endeavour, a career opened to talent, and every individual in the profession would have free scope to elevate himself to any height which his talents and industry could enable him to attain. A further advantage of such a system is that all members so entering the profession, being on their introduction fully and equally proved as to their competency, all pretext for subjecting them to further ordeals would be removed, and individuals would be free to practise wherever their own interests or inclinations might determine. Such improvements in the profession would realize all that the most sanguine could desire.

### IV. *Report of the Committee appointed to consider the Nature, Extent, and Evils of Quackery.*

The great extent and importance of the subject, the difficulty of acquiring extensive and correct statistical information in regard to it, and the present unsettled state of the internal arrangements of the medical profession itself, combined with the natural and almost necessary differences of opinion as to the precise measures which it might be desirable to propose—these and other reasons, unnecessary to allude to, have prevented your Committee from coming forward and presenting you with a formal Report.

They are induced, however, to hope that you will not consider that they have wholly neglected the duties intrusted to their care, and that in the statement which accompanied the account of the proceedings of the last Anniversary, (put forth, it is true, upon individual responsibility, but with the sanction and assistance of the members of the committee generally,) you will admit that some additional information has been obtained.

The *machinery of Quackery* was there partially exposed; and the reasons why it should be legally suppressed, with the popular and not unfrequent professional objections to all legislative interference, were freely canvassed, and, we trust, satisfactorily replied to.

The *interests of Government* in empiricism were also, for the first time, accurately investigated; and it was shown, from unquestionable authority, that the revenue from quackery is less than 50,000*l.* a year. This fact is not without importance, since one of the most tenable and practical objections urged by the opponents to all active measures, was the great amount of the government profits. The true statement of the case is, however, now before you; and it is evident, whatever other obstacles may exist, that the one now alluded to can no longer be regarded as either insuperable or formidable.

The actual state of existing anti-empirical enactments has also been examined, and, while it cannot but be admitted that much of the present respectability and standing of the medical profession is to be attributed to the legal penalties and restrictions which have from time to time been enforced against ignorant and unqualified practitioners, (the number of whom, great as it still is, has in consequence been materially diminished,) it is at the same time equally clear that no efforts have yet been made to prevent or even to curtail the irresponsible and indiscriminate circulation of *medicines;* but that, by the stamp and patent regulations, this glaring abuse is legalized and encouraged; and, from the unequalled facilities which now exist for advertising, an injury upon the public health is inflicted, far greater than could ever be the case from the strictly personal, though unqualified practice of physic.

Procuring the abolition of the stamped and patent medicines, at least in their present irrestricted form, should therefore be the great and leading indication of our efforts at the present moment; and your Reporter is convinced that the greatest if not the only serious obstacles to its accomplishment are the inertness and apathy of the medical profession.

That medical men are alone competent to expose the evils of empiricism, and that, as legalized guardians of the public health, they are, *ex officio,* as well as morally, bound to do their utmost for its suppression, are axioms so palpably correct, that they need no arguments for their support; and, while we should be justified in coming forward and demanding redress, upon the grounds that our privileges and constitution are grossly infringed, we are still more strongly called upon to exert ourselves for the benefit and protection of those, the conservation of whose health is the very object of our corporate existence.

It is of little importance to determine whether, as a profession, we should gain or lose, in a pecuniary sense, by the suppression of quackery, since the public are naturally and justly but little interested in the amount of our private emoluments; but knowing, as we do, the injurious effects of empiricism upon the national health, it *is* important and unquestionably our duty that we should, irrespective of all personal interests, stand boldly forward and denounce it, while we exert our influence, both in public and private, to check and resist its encroachments.

Our best claims upon public confidence and respect should rest, not upon a timid and cringing servility to popular prejudices, or upon a morbid, selfish sensibility, which dreads collision with existing interests, hostile as we believe these to be to the general welfare, but upon a firm and disinterested insisting upon what we know to be essential to the public good—upon a temperate but decided assertion of our medical authority, and upon a uniform refusal to sacrifice our professional character to public ignorance and caprice.

The anomalous condition in which we are now placed, the indignities to which, as a profession, we have been exposed by the public authorities, the cheap estimation in which we are held, and the open and successful competition which impudence and avarice have so long been able to maintain against us, are mainly to be attributed to the want of a more honorable and independent spirit among ourselves, to the not attaching sufficient weight and importance to the office we hold, to the frequent absence of unity and sincere cooperation in our ranks, and to the too eager pursuit by many of individual benefit, at the expense of the body to which they belong. Until we are true to ourselves, we may in vain expect the full harvest of public confidence and esteem; and this precept remains still unfulfilled, so long as we silently submit to the gross encroachments of modern quackery, and while we have no means of expelling from our ranks all those who sacrifice the best interests of their profession at the shrine of a selfish and ignominious cupidity.

Medical reform can only be securely effected by the exertions of medical men; and it is idle to expect assistance from without, unless we show a willingness to purify and a power to defend ourselves. Instead of vaguely descanting upon our grievances, let us actively unite for their removal, and rest assured there is no reasonable limit to the good which might then be accomplished. The present Association furnishes us with an engine of no ordinary magnitude and influence; and, while we hope that the great and leading questions which more directly affect the public welfare and our corporate purification, will ever engage its primary and earnest attention, we trust that it will also constitute a permanent *court of honour*, excluding from amongst us all whose conduct is at variance with the higher moral interests of a profession, whose dignity and usefulness we are pledged by every honorable feeling to uphold.

The question of medical quackery is at this moment engaging the serious attention of the French government, and very stringent regulations may be shortly expected. The British Medical Association is also occupied in collecting information upon the subject, in conjunction with a plan of general medical reform. Let us not, then, withhold our cooperation, but let each member feel himself personally interested in the struggle, and pledged to furnish his mite of information and suggestions to those who may be more particularly intrusted with the labour of revision and arrangement.

It is only by a careful digest of a large aggregate of facts and opinions that satisfactory results can be obtained, and there is no subject more deserving of the combined talent and energies of the Provincial Medical and Surgical Association, and in the right settling of which so many important interests are involved, than the one to which we are now directing your attention.

For the reasons already alluded to at the commencement of this report, your committee have refrained from submitting, for your approval, any definite measures for the suppression of empiricism, being anxious, if possible, first to secure the united cooperation of the members, before differences of opinion are excited by the discussion of secondary details. They may, however, be permitted to remark, that quackery in its actual and grosser manifestations is capable of being as effectively interfered with as abuses of many other descriptions; and, though the hope of its *utter* extinction may justly be regarded as chimerical, no reasonable doubt can be entertained by those who have considered the facts of the case, that its present daring aspect and alarming prevalence, may be most materially modified and controlled by well-directed legislative prohibitions.

To accomplish so desirable a result, it is only necessary for the medical profession of Great Britain zealously and cordially to unite, and, by doing so they could not fail to secure the attention and support of Government, as well as to enlist in their favour the common sense and correct feeling of every conscientious and enlightened individual.

We would also beg leave to observe, that reiterated experience too plainly proves that all other means of a less direct or more prospective character must ever prove comparatively inert and unavailing; but if aided by efficient legislative protection, they would necessarily tend to disabuse the public mind of those prejudices and misconceptions on which the quack so successfully trades.

Among the more important of these accessory measures may be enumerated popular lectures on empiricism, by which some are convinced and others shamed out of their delusions; also the circulation of tracts, unfolding the iniquities of the system, and demonstrating its irrationality; and lastly, that more limited but perhaps more effectual exposure of its dangers and inconsistencies, which our daily intercourse with its victims permits, but which we are too apt to neglect, either from morbid sensitiveness, moral cowardice, or the shortsighted promptings of self-interest.

In conclusion, let us resolve no longer to tolerate quacks among ourselves; but by a uniform and unflinching attachment to truth—by unwearied efforts for the acquisition of knowledge and its enlightened application for the removal and alleviation of human suffering—by a constant willingness to acknowledge and eradicate abuses, wherever they may exist,—let our conduct and bearing, individually and collectively, be such as at all times to entitle us to the sympathies and support of every upright and intelligent mind.

### V. *Petition to Parliament for Reform of the Profession.*

That this petition emanates from a body of medical practitioners, comprising nearly twelve hundred members, and entitled, the Provincial Medical and Surgical Association.

That the members, being derived from every county in England, and the Association open to all regular medical practitioners who desire admission, it may be considered as representing the provincial medical faculty of this portion of the United Kingdom.

That your petitioners naturally feel a deep interest in the welfare and respectability of the profession to which they belong, and have long deplored the manifold evils to which it has been subjected by the want of a suitable political organization, such as should both ensure the professional competency of all who enter it, and give full legal protection to all who bring to it the ordained qualifications.

That your petitioners hailed with peculiar satisfaction and gratitude the disposition manifested by Parliament, in 1834, to take this important subject into consideration, and rejoiced in the appointment by the House of Commons of a Committee to enquire into the general state of the profession, assured that due enquiry alone was needed to demonstrate the necessity of legislative intervention.

That your petitioners marked, with high approval, the diligent scrutiny so ably conducted by this committee, and have anxiously awaited the publication of the minutes of the evidence taken before it. But five years having elapsed since the committee completed its enquiries, without the whole minutes of evidence being yet published, or any report of the committee presented, your petitioners feel that they should fail in the duty which they owe to their profession if they longer delayed appealing to the legislature in its behalf.

It was contemplated by your petitioners to solicit the House of Commons for enforcement of their resolution of the 13th of August, 1834, which called on the Committee of Medical Enquiry for the Minutes and Report. But circumstances have occurred which render your petitioners less anxious respecting the proceedings of this committee, it appearing to your petitioners that the objects sought by that enquiry may be attained through other more direct and effectual means.

Your petitioners beg leave to represent that the main requisite and only stable foundation for any sound system of medical polity is to establish an adequate and uniform education for the whole profession, so that all who enter it shall pass through the same course of preliminary and medical instruction, be tested by the same examinations, and, when approved, entitled to the same privileges. The natural unity of the profession imperatively demands this consolidation, there being no more preposterous or mischievous anomaly than that presented by the existing state of the medical institutions of this kingdom, where practitioners of physic issue from no less than sixteen separate sources, differing from each other in the course of education enjoined, the qualifications required, the examinations by which the qualifications are tested, and the privileges conferred.

Finally, your petitioners, confident that the time is arrived when the intervention of Parliament is imperatively called for to give to the medical profession a sound legal constitution, and deeply impressed with the conviction that an adequate and uniform education, with community of rights and privileges, constitutes the only sure basis on which to found such constitution, humbly beg that the necessary legislative enactments may be passed for establishing in each of the three divisions of the kingdom one superintending body, founded on the same principles and governed by similar regulations, through whose examination and by whose licence alone, shall admission to the profession be in future attained.

VI. *Petition to Parliament respecting Vaccination.*

That this Association consists of nearly twelve hundred members, including a great proportion of the physicians and surgeons of eminence, practising in the various cities and towns of England, most of whom, during the whole of their professional lives, have bestowed much of their attention in studying the qualities of human smallpox and cow-smallpox.

That at their last anniversary meeting, held in July, 1838, at Bath, a section was appointed to enquire specifically into the present state of vaccination.

That the section so appointed hath recently presented its Report to the meeting held at Liverpool, containing information of an highly important character, and well deserving the attention of your Honorable House.

That your petitioners have peculiar satisfaction in stating that the result of their enquiries has thrown much light on the nature of *variolæ vaccinæ*, and that Dr. Jenner's opinion, that it was an affection of a true variolous character, has been demonstrated by historical evidence, as well as by direct experiment, human smallpox having been recently communicated to the cow by inoculation, and the result having been the production of a *variolo-vaccine* lymph, possessing all the properties of the original vaccine disease.

That this direct confirmation of a great doctrine adds infinite value to the original discovery, by explaining alike the nature and the degree of protection that may be derived from perfect vaccination, which is, in short, to use the language of the discoverer himself, to impregnate the constitution of man with smallpox in its mildest, instead of its pestilential and fatal form.

That the diffusion of this truth may be made subservient to the best purposes, and, with the aid and countenance of your Honorable House, be rendered highly instrumental to the preservation of human life.

That your petitioners have learned, by the concurrent testimony of a very large portion of their members, that cow-smallpox, if duly and carefully communicated, has an enduring influence in protecting the constitution; that while they admit that this protection is not in all cases complete, they have unquestionable proof of its being capable, if generally and properly employed, of mitigating, controlling, and, they might almost say, of extinguishing smallpox in any district.

That they have further learned that, while vaccination has been imperfectly and insufficiently employed, in many places, smallpox has been and continues to be diffused in a manner highly detrimental to the health and safety of the community.

That, before suggesting any measure for the more efficient diffusion of vaccination, they would specially implore your Honorable House to take measures for regulating the practice of smallpox inoculation; and they are induced to urge this prayer of the petition with greater earnestness, because they have ascertained that such practice has been abandoned by almost every respectable medical man in the kingdom, from a disinterested conviction that it is uncalled for and dangerous, and ought to be universally superseded by vaccine inoculation.

That the abandonment of smallpox inoculation by medical gentlemen has led ignorant and illiterate persons to take up the practice, whereby the disease has been widely disseminated throughout the country, and that many deaths have been the consequence.

That your petitioners humbly beg leave to represent to your Honorable House the extreme necessity of restraining such proceedings; they do not ask for a positive enactment to forbid smallpox inoculation entirely, though they think there are sufficient grounds for so doing; but they pray that it may be enacted that a practice, always dangerous and very frequently fatal to the subject of it, as well as to the community at large, shall not be permitted to be followed by any one who has not been duly qualified to exercise the profession of medicine or surgery; that such restriction, in their opinion, would be wise and salutary at all times, but is more especially demanded now that a mild and efficient substitute may be brought within the reach of every member of the community. To accomplish this latter object your petitioners humbly submit that certain regulations ought to be adopted. They have ascertained that during recent epidemics, a very large proportion of the lower classes, both in towns and among the rural population, have been found totally unprotected; that in one city in the south of England, five hundred persons perished from this cause in the course of last year; and that in another place, on the breaking out of smallpox, the whole of the children under nine or ten years of age were exposed to its ravages, which were only checked by prompt and extensive vaccination.

That these and many similar facts have convinced them that at this time there is no sufficient provision for the vaccination of the poor in this kingdom; that the practice, as offered to the poor at our public institutions in towns, as well as by private individuals, is by no means adequate to the wants of our greatly increasing population.

That it appears to your petitioners to be the duty of the State to remedy this great evil, by appointing regularly-educated vaccinators, with suitable salaries, in districts sufficiently numerous to embrace the whole of the poor population of the country, and who shall offer gratuitous vaccination at stated periods to all within their bounds, keeping accurate registers of their proceedings, and communicating regularly with the national vaccine establishment.

That under such a system, duly organized and vigilantly executed, your petitioners have the strongest reason for believing that smallpox would be effectually restrained, and soon become almost unknown.

### VII. *Important Experiments in Vaccination.*
### Performed by Robert Ceely, Esq. of Aylesbury.

Mr. Ceely exhibited several original and beautifully executed coloured drawings, illustrative of the following subjects:

1. Two successful inoculations, in different stages, of young stirks, with smallpox matter.

2. Numerous vesicles, in different stages, in several human subjects, chiefly children, produced by the lymph obtained from these inoculations.

These drawings were intended to illustrate the experiment alluded to in the admirable report of the vaccination section, read at the meeting on the same day by Dr. Baron; and in which it was declared that there could now be no doubt as to the correctness of the doctrine first announced, and always maintained by Dr. Jenner, that human and vaccine variolæ are identical, the latter being merely a mild modification of the former.

After minutely describing these interesting and well-executed drawings, Mr. Ceely remarked that he had several times inoculated cows, in various ways, with smallpox virus, in different stages, either without success, or with the production of *tubercles* or pimples, without lymph—that these two successful inoculations were performed in the beginning and middle of February last, at a time when small-pox was prevailing in Aylesbury—and when he had invested three other cows with blankets from the bed of a smallpox patient, and employed a man, recently convalescent from smallpox, frequently to walk among and handle all the animals under experiment.

These experiments, he stated, were witnessed by five medical men, a veterinary surgeon and his assistant, all of Aylesbury, who were perfectly satisfied with the manner in which they were conducted, and highly gratified with the unexpected results

The two animals, on which the above experiments succeeded, were not more than twelve months old. The parts selected for inoculation were the sides of the labia pudendi, and adjacent vicinity.

In the first animal, fourteen points (the teeth of a new comb), well charged with fresh virus of 7th or 8th day of variola discreta, were inserted into seven punctures in the left side of the labium pudendi, from which only one vesicle was produced; the other punctures proving tubercular or papular. Until the *tenth* day, there was some doubt as to the probable character of that which ultimately became perfect: but on *this* day, clear pellucid lymph was taken on points, and immediately inserted into the arms of several children. The animal, on the *ninth* day after variolation, and *before* a perfectly vesicular appearance was observed, was *vaccinated* with recent dry lymph from a child in eleven punctures on the right side of the labium pudendi. On the *fifth* day after vaccination, (and thirteen of variolation,) when *every vaccine* puncture had succeeded, lymph was taken from two or three of the vaccine vesicles, and also inserted into the arms of some children.

The *variolous* vesicle, and the *vaccine vesicles* ran their course together; the former attaining its acmê on the fifteenth day; the latter, theirs, of course, on the seventh day, and were smaller. The lymph from the variolous vesicle, and that from the vaccine vesicles, produced in the human subject the true vaccine; the former, for a short time, appearing less active than the latter; but finally, losing all traces of disparity.

In the second experiment, recent liquid smallpox virus, (seventh or eighth day variola discreta,) in *capillary tubes* was used. The punctures were first deluged, each with the contents of one or two tubes; and new points of the same materials as used in the other experiment, well charged from the same patient, on the same day, were cut off short, and left in the wounds till the next day. Eight punctures were made—four above, and four upon the back of the left labium pudendi. All had risen on the fifth day; but only the four upon the *back* of the labium advanced, and produced pellucid lymph; the others near the ischium and perineum, for a time tubercular or papular, gradually subsided. Lymph was taken from one of the four vesicles on the sixth, eighth, ninth, and tenth days; the disease declining on the eleventh day. This lymph, too, was inserted into the arms of children, and produced, especially that of the eighth day, vaccine vesicles of a remarkably fine character. Many hundred children and some adults, at Aylesbury, Cheltenham, and the Smallpox and Vaccination Hospitals, London, have been vaccinated from this source. The efficiency of the lymph has been fully tested by infection and inoculation, both of which it has resisted. Both *stirks* have resisted careful and repeated *reinoculation* and *revaccination.*

3. Experiments in *retro-vaccination*, or vaccination of the cow from the human subject, and of the effects of the resulting lymph again transferred to the human subject.

Mr. Ceely remarked, that although the most successful vaccinations of the cow are effected by the reiterated manipulations of the milkers—yet the young stirk can be vaccinated with comparative facility with lymph recently obtained from the milch cow. It is, however, not so easy to *retro-vaccinate* from the human subject, as he had learned from many experiments. When successful, in general, the disease, thus induced in the young animal, arrives at its acmé between the tenth and eleventh days—the normal course of the natural disease in the milch cow. The *renewed* lymph thus produced seems to have undergone a change—for if taken from the seventh to the tenth day, and returned to the human subject, it rarely produces a vesicle at the acmé till the eleventh, twelfth, or thirteenth days —and then often smaller than ordinary, but in about two or three removes from the cow it recovers its previous activity. As in man, so in the cow, revaccination is unsuccessful at an early period.

4. The artificial vaccine vesicle in dogs, and an occasional attendant vesicular eruption. The dog, it is well known, is very susceptible of vaccination; the vesicle appears to run a similar course as that in the cow and in man; but is manifestly smaller, yielding much less lymph. This animal, too, is liable to a consequent vesicular pemphigoid eruption, resembling, in miniature, what is occasionally noticed in infants about the tenth or fourteenth days of vaccination.

5. The natural (and genuine) variolæ vaccinæ on the teats and udder of a white milch cow, exhibiting vesicles of different sizes in different stages,—entire, incrusted, or broken.

6. The *White Pock.*—This, though in general a mild and unimportant disease, has, to an inexperienced observed, many features in common with the true cow-pock, especially towards its close and period of incrustation; but Mr. Ceely showed, that by a little attention, particularly in the early stage, they might be readily distinguished. It was only in the stage of incrustation that an error might be committed. He remarked that it might be said that the *white pock* and the true *variolæ vaccinæ* bore the same relation to each other in elementary character, progress, maturation, and decline, as varicella does to human variolæ.

7. The *Yellow Pock.*—A pustular disease, resembling, at first, ecthyma vulgare, and ultimately assuming an impetiginoid character.

8. The *Blue Pock.*—A vesicular disease, often putting on the appearance of impetigo.

These varieties of the spurious disease depicted on the teats and udders of the milch cow, frequently occur, at all seasons, from common causes, especially in heifers on their first milking, are often intercurrent, and were taken from subjects inspected in the same dairies where the true cowpock coexisted. The latter would appear to be not an unfrequent visitor of the numerous dairies in the rich vale of Aylesbury, during the autumn, winter, and spring; and Mr. Ceely seems to have made diligent use of the opportunities thus afforded him of observing this and other eruptive diseases of cows. It is evident he has been at great pains, and considerable expense in collecting and illustrating the many facts we have here merely glanced at, and we are glad to hear that it will not be long before they are made public in such minute detail as their novelty and interest demand.*

* Dublin Medical Press. Aug. 1839.

